# Ocular Complications of Zygomatic Dental Implants: A Systematic Review

**DOI:** 10.7759/cureus.67535

**Published:** 2024-08-22

**Authors:** Moshe I Weber, Elliot Koschitzki

**Affiliations:** 1 Ophthalmology, State University of New York Downstate Health Sciences University, New York, USA; 2 Dentistry, Long Island Implant and Cosmetic Dentistry, New York, USA

**Keywords:** orbital penetration, dental implant, orbit, eye, complication, zygomatic implant

## Abstract

Zygomatic implants are a form of dental implant that anchors in the zygomatic bone with potential for complications to the eye and orbit. This article presents a systematic review of the literature regarding ophthalmological complications of zygomatic implants to familiarize ophthalmologists with the potential complications and their treatment options. The review was conducted using the Preferred Reporting Items for Systematic Reviews and Meta-Analyses (PRISMA) guidelines. Various searches using PubMed, Elsevier, and Google Scholar were used to search for articles using search terms including ‘zygomatic’, ‘dental’, ‘implant’, ‘complications’, ‘eye,’ and ‘orbit.’ Exclusion criteria included articles that were unavailable in English or written before 1980. Twenty-four articles were included in this review: nine case reports, one case series, nine cohort studies, one randomized controlled trial, and four review articles. The most common complication was infraorbital paresthesia, followed by intraoperative orbital penetration. Other complications included implants placed into the orbit, orbital hematomas, extraocular muscle damage, diplopia, subconjunctival hemorrhage, periorbital fistulae, infraorbital rim infections, and orbital emphysema. Of 41 cases, which included outcomes, 10 patients required further procedures, and five patients had irreversible damage. It is important for both oral and maxillofacial surgeons and ophthalmologists to recognize these complications for proper coordination of care and treatment.

## Introduction and background

Dental implants are a common dental procedure used to create support for prosthetic teeth. Although they are typically anchored by titanium rods placed in the maxilla, in cases of severe maxillary atrophy, there can be insufficient bone to create a stable attachment point for the implants [1]. In the past, bone grafts were used to increase maxillary bone and create a stable implant. However, bone grafts require additional procedures for bone procurement, increasing morbidity, and require waiting months before implants can be placed [2]. One alternative is the zygomatic implant. In zygomatic implants, additional longer zygomatic fixtures are placed to improve the stability of the implant and decrease reliance on bone grafts [1]. These fixtures run through the maxilla to the anterior portion of the zygomatic bone, passing near the floor of the orbit [1]. Although this technique has been in use for over 20 years, it remains a highly specialized technique due to the high degree of technical difficulty [3]. One of the sources of this difficulty is the small area of dense zygomatic bone the implant must be placed in and its close proximity to the orbit, resulting in the potential for orbit penetration and ocular complications [1]. Recently, computer-generated 3D models and dynamic navigational systems are being developed to make zygomatic implants easier to place, resulting in increased popularity of the procedure [3]. 

Although the theoretical complications of orbit penetration are often mentioned in the literature, the current literature of ophthalmologic complications of zygomatic implants consists of individual case reports and incidental findings in studies designed to find survival rates of zygomatic implants. This paper seeks to perform a systematic review of the current literature on eye and orbit complications from zygomatic implants.

## Review

Methods

**Figure 1:**
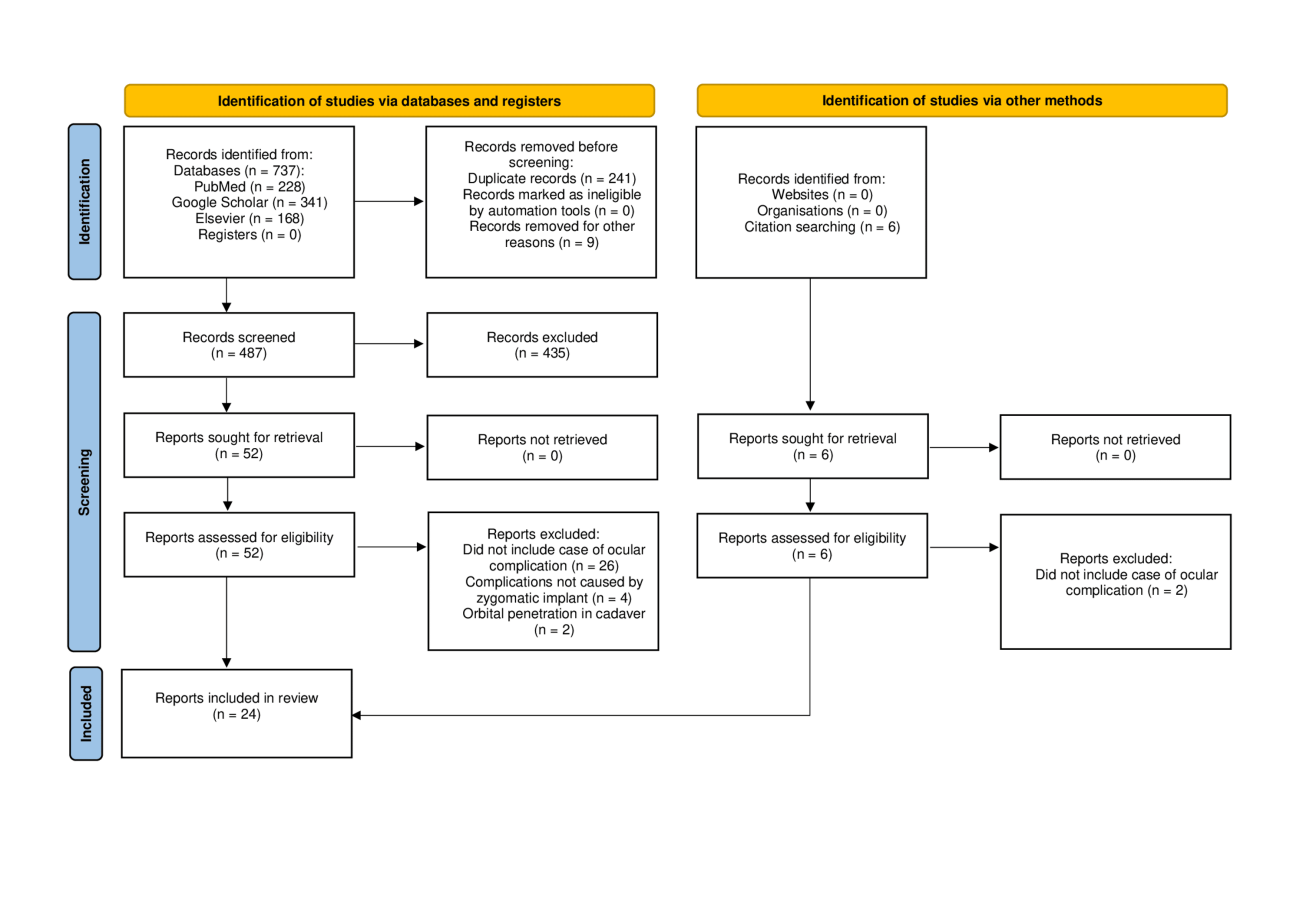
PRISMA flow diagram. PRISMA: Preferred Reporting Items for Systematic Reviews and Meta-Analyses. Source: Haddaway et al. [4].

This study was performed following the Preferred Reporting Items for Systematic Reviews and Meta-Analyses (PRISMA) guidelines. PubMed, Elsevier, and Google Scholar were used to search for articles written between 1980 and 2024 using the terms ‘zygomatic’, ‘dental’, ‘implant’, ‘complications’, ‘eye,’ and ‘orbit’ (last searched April 2024). Articles were screened out using title and abstract, followed by a full-text review by two reviewers to select articles to be included (Figure 1). Forward and backward citations were then checked for additional articles. The inclusion criteria were any articles that reported cases of damage to the eye or orbit as a result of zygomatic dental implants or implantation. Case reports, cohort studies, randomized controlled trials, and review studies were included. The exclusion criteria were articles not available in English and those written before 1980. Perizygomatic infections or complications resulting from concomitant tumor removal were not included in this review as ocular complications of zygomatic implants. Articles were analyzed for ocular complications, including the year of the study, type of complication, symptoms associated with the complication, long-term outcome, and if the patient received a second ipsilateral zygomatic implant.

Results

Twenty-four studies were found to contain cases of eye or orbit complications from zygomatic dental implants: nine case reports [5-13], one case series [14], nine cohort studies [15-23], one randomized controlled trial [24], and four review articles [25-28] (Table 1). After accounting for duplicates, 49 distinct patients were identified as having suffered ocular complications. Twenty-seven cases of infraorbital paresthesia, 11 cases of orbital penetration, four cases of orbital implantation, five cases of subconjunctival hemorrhage, four cases of periorbital hematoma, three cases of periorbital fistula formation, three cases of lateral rectus damage, two cases of inferior oblique damage, two cases of intraorbital hematoma, one case of recurrent orbital rim infection, one case of infraorbital nerve damage, one case of orbital compression syndrome, and one case of orbital wall defect with orbital emphysema. Symptoms included bleeding, restricted ocular range of motion, diplopia, esotropia, and proptosis, along with pain and swelling. Of the 49 patients who experienced ocular complications, outcomes were reported for 41 of them. Outcomes were generally favorable, with 88% (36/41) of patients experiencing full resolution of symptoms. Ten patients required surgical treatment, most commonly implant removal (4/10). Four patients experienced a permanent range of motion deficit and one patient experienced permanent sensory loss. 

**Table 1:** Articles included in this review.

No.	Author, year	Article type	Title	Complication	Symptoms	Treatment	Outcome	Second ipsilateral implant
1	Mavriqi et al., 2021 [5]	Case Report	Zygomatic implant penetration to the central portion of orbit: a case report	Zygomatic implant penetrating the central part of the orbit	Diplopia, difficulty moving the eye, pain, swelling, and paresthesia	Removal of the implant	Full resolution	No
2	Van Camp et al., 2018 [6]	Case Report	Intraorbital hemorrhage following a secondary intervention at integrated zygomatic implants: a case report	Orbital hematoma following zygomatic implant overhang removal resulting in orbital compression syndrome	Proptosis, pain, and severe swelling	Emergency surgical drainage, with Penrose drains left in place for two days	Full resolution	Yes
3	Krauthammer et al., 2017 [7]	Case Report	Extraocular muscle damage from dental implant penetration to the orbit	Zygomatic implant penetrating lateral portion of the orbit abutting the lateral rectus and inferior oblique	Bleeding from the lateral canthus, restricted abduction, diplopia, and subconjunctival hemorrhage	Removal of the implant, followed by adhesion removal and Hummelsheim procedure one month later	Restricted abduction and elevation accompanied by diplopia	Yes
4	Dawood et al., 2017 [8]	Case Report	Management of extraoral complications in a patient treated with four zygomatic implants	Recurrent infraorbital rim infections	Pain and swelling of infraorbital rim	Implant apical resection	No long-term follow-up reported	Yes
5	Garcia et al., 2015 [9]	Case Report	Bilateral cutaneous fistula after the placement of zygomatic implants	Inflammatory lesions with fistulation on the lateral aspects of the orbit	Suppuration and spontaneous drainage, pain, and swelling	Fistulectomy after failing antibiotic treatment	Contralateral recurrence. Full resolution of both	Yes
6	Tran et al., 2018 [10]	Case Report	Zygomatic dental implant induced orbital fracture and inferior oblique trauma	Orbital penetration with orbital implantation, hematoma, fibrosis, and damage to the inferior oblique	Periorbital swelling, inability to open the eye, diplopia, hypotropia, limited adduction, lower lid retraction	Implant removal and exploratory adhesion removal, followed by strabismus surgery six months later	Orthotropic alignment without diplopia following strabismus surgery	Yes
7	Chang et al., 2019 [11]	Case report	Delayed orbital emphysema mimicking orbital cellulitis: an uncommon complication of dental surgery	Orbital emphysema with orbital wall defect	Orbital and periorbital swelling, proptosis	None	Spontaneously resolved over two weeks	No
8	Cikatrics et al., 2008 [12]	Case Report	Iatrogenic lateral rectus transection secondary to dental implantation surgery	Orbital penetration with implant placement into the orbit and transection of the lateral rectus	Subconjunctival hemorrhage, esotropia, diplopia, swelling	Reattached viable lateral rectus and botulinum toxin injections into medial rectus over nine months, further strabismus surgeries	Permanent ocular range of motion deficits	No
9	Topilow et al., 2020 [13]	Case Report	Extraocular muscle injury in zygomatic implant placement: a case report, review of the literature, and simple maneuver for avoidance	Orbital penetration and partial transection of the left lateral rectus	Subconjunctival hemorrhage, abduction deficit, diplopia, esotropia	Implant placement was aborted. Initially, prism glasses were used; however, after worsening diplopia strabismus, surgery was performed	Improved primary gaze alignment, persistent esophoria, range of motion deficits, and diplopia on rightward gaze	No
10	Tzerbos et al., 2016 [14]	Case Series	Complications of zygomatic implants: our clinical experience with four cases	Aseptic necrosis and fistulation of the zygomatic-orbital area	Not reported	Fistulectomy and implant apical resection	Full resolution	No
11	Davó et al., 2010 [15]	Cohort Study	Immediate function of four zygomatic implants: a one-year report of a prospective study	Orbital cavity penetration	None	None	Full resolution	Yes
12	Davó et al., 2013 [16]	Cohort Study	Prostheses supported by four immediately loaded zygomatic implants: a three-year prospective study	Orbital cavity penetration	None	None	Full resolution	Yes
13	Davó et al., 2015 [17]	Cohort Study	Five-year outcome of cross-arch prostheses supported by four immediately loaded zygomatic implants: a prospective case series	Orbital cavity penetration	None	None	Full resolution	Yes
14	Hinze et al., 2013 [18]	Cohort Study	Zygomatic implant placement in conjunction with sinus bone grafting: the 'extended sinus elevation technique'. A case-cohort study	Orbital penetration with implant placed into the orbit penetrating orbital fat	Detected on postoperative cone-beam computed tomography scan. No symptoms	Implant removal	Full resolution	Not reported
15	Duarte et al., 2007 [19]	Cohort Study	The establishment of a protocol for the total rehabilitation of atrophic maxillae employing four zygomatic fixtures in an immediate loading system--a 30-month clinical and radiographic follow-up	Two cases of orbital cavity penetration	Hematoma 'at the level of the scleral and subconjunctival tissue'	None	Spontaneous resolution over two weeks	Yes
16	Kahnberg et al., 2007 [20]	Cohort Study	Clinical evaluation of the zygoma implant: three-year follow-up at 16 clinics	Infraorbital nerve damage	Paresthesia	None	Paresthesia remained	No
17	Wu et al., 2015 [21]	Cohort Study	Restoration of oral function for adult edentulous patients with ectodermal dysplasia: a prospective preliminary clinical study	Four cases of periorbital hematomas	None	None	Spontaneous resolution in all cases over one week	No
18	Fernandez et al., 2013 [22]	Cohort Study	Zygomatic implants for the management of the severely atrophied maxilla: a retrospective analysis of 244 implants	Infraorbital paresthesia	Not reported	Not reported	Not reported	Not reported
19	Davó et al., 2024 [23]	Cohort Study	Long-term survival and complications of quad zygoma protocol with anatomy-guided approach in severely atrophic maxilla: a retrospective follow-up analysis of up to 17 years	Two cases of orbital penetration Five cases of infraorbital paresthesia	None	none	Full resolution in cases of orbital penetration	Yes
20	Davó et al., 2018 [24]	Randomized controlled trial	Immediately loaded zygomatic implants vs. conventional dental implants in augmented atrophic maxillae: one-year post-loading results from a multicenter randomized controlled trial	One case of periorbital swelling One case of periorbital infection and fistulation 21 cases of infraorbital paresthesia	Not reported	Not reported	Full resolution in all cases	Not reported
21	Chrcanovic et al., 2016 [25]	Review article	Survival and complications of zygomatic implants: an updated systematic review	Inferior orbital paresthesia	Not reported	Not reported	Not reported	Not reported
22	Wang et al., 2015 [26]	Review article	Reliability of four zygomatic implant-supported prostheses for the rehabilitation of the atrophic maxilla: a systematic review	Orbital cavity penetration	Not reported	Not reported	Full resolution	No
23	Varghese et al., 2021 [27]	Review article	Rehabilitation of the severely resorbed maxilla by using quad zygomatic implant-supported prostheses: a systematic review and meta-analysis	Orbital cavity penetration	Not reported	Not reported	Not reported	Not reported
24	Tavelli et al., 2022 [28]	Review article	Survival and complication rate of zygomatic implants: a systematic review	Two cases of orbital cavity penetration	Not reported	Not reported	Not reported	Not reported

Discussion

Dental implants are a common procedure used in clinical practice to aid in the placement of dental prostheses. While most maxillary dental implants are placed in the alveolar ridge, edentulous patients experience bone atrophy and resorption, often leaving the maxilla unsuitable to support implants [1]. As an alternative, the implantation of longer implants anchored in the zygomatic bone is an option that is increasing in popularity [3]. These implants allow for immediate prosthesis placement and have a high survival rate ~98% [27]. However, due to the technical difficulty of implantation, small area of dense zygomatic bone, and close proximity to the orbit, these implants come with the risks of improper drilling and implant placement, including the risk of rare but serious ocular complications. Orbital hematomas, perihematomas, emphysema, extraocular muscle damage, infraorbital infections, infraorbital nerve damage, and orbital fistulation, along with permanent ocular range of motion defects, diplopia, and esotropia, have been reported. 

Orbital Penetration

The most severe ocular complication of zygomatic implant placement was orbital penetration. Ten cases of orbital penetration have been reported, with 30% (3/10) cases resulting in permanent ocular range of motion deficits [7,12,13]. Due to the superolateral angle of drilling up from the maxilla, the orbit can be breached during drilling of the osteotomy in the inferolateral portion [15]. Minor 'skirting' breaches of the orbital floor or lateral wall that occur by obliquely drilling through the superomedial portion of the zygomatic bone usually result in no long-term ocular consequences [15,18,19]. More medial or deeper penetrations of the orbit can result in damage to the lateral or inferior rectus muscle and cause intraorbital adhesions resulting in ocular range of motion defects [13]. Most cases of orbital penetration were discovered during or immediately after surgery [7,10,12,13,15]. However, one case was found only on routine postoperative cone beam computed tomography (CBCT) [18]. Orbital penetration was followed by orbital implant placement in 50% (5/10) of cases. Orbital implantation resulted in damage to the lateral rectus and/or inferior oblique in 60% (3/5) of cases, resulting in ocular range of motion loss, esotropia, or diplopia despite implant removal [3,7,9]. However, some cases can have minimal consequences [18]. There have been no reported cases of a ruptured globe; however, considering multiple cases of extraocular muscle damage have been reported, globe rupture remains a risk.

To decrease the risk of orbital penetration, Topilow et al. [13] suggested using a stainless steel plate (Bausch and Lomb E1069) placed in the inferior conjunctival fornix to protect the orbit and provide the surgeon immediate tactile feedback if the orbit is breached. Additionally, using the 'extrasinus technique' as opposed to an 'intrasinus technique' may allow for better visualization of the drill during the osteotomy preparation and decrease the risk of orbital penetration. The intrasinus technique typically involves starting the osteotomy from the residual maxillary alveolar ridge, transversing the maxillary sinus until penetration of the zygomatic bone [1]. Due to the fact that the zygomatic implant transverses the maxillary sinus internally, visualization of the drill and implant is severely compromised. However, the 'extrasinus technique' utilizes a diamond barrel bur to create the initial osteotomy along the lateral wall of the sinus prior to entering the zygoma enabling better visualization [29]. Zielinski et al. [30] suggested the use of a zygomatic orbital floor (ZOF) classification system to reduce the risk of orbital penetration. By measuring the difference in height from the most lateral to most medial portion of the zygomatic bone, practitioners can have a measure of the degree of undercut of the orbit to better plan surgery. Alternatively, dynamic navigation systems (DNS) have been proposed to decrease the difficulty of zygomatic implantation [3]. By mapping current drill tip location to pre-surgically mapped 3D models, exact real-time drill location and direction can be determined. This allows the surgeon to control angulation during drilling to avoid the orbit. While these technologies are promising and becoming more available, no studies have determined their ability to decrease the risk of orbital penetration. Routine use of post-implantation CBCT may be useful in finding asymptomatic orbital penetration or implantation [15].

The incidence of orbital penetration during zygomatic implant placement is difficult to determine. Most cases are found as case reports, and no sufficiently large study has been done to determine its incidence. Estimates therefore vary wildly with (5.9%-0.66%) [22,27,28]. However, these are likely overestimates as studies that contain at least one case of orbital penetration are more likely to be included: leaving out the majority of studies which report none. Using the data obtained in a large-scale systematic review of the survival rates of zygomatic implants, of the 1,975 patients in cohort studies and randomized controlled trials for which complications were reported, two cases of orbital penetration were documented indicating the incidence to be roughly 0.1% [27]. However, this method likely underestimates the incidence as these studies were not designed for analysis of orbital penetration, possibly resulting in some cases going unreported as complications. 

Management for orbital penetration should be decided on a case-by-case basis. CBCT or CT is performed to visualize the location of the implant and orbital structures [7,10,12,13]. If the implant was placed in the orbit, prompt removal was performed in all (5/5) cases. Other than removing the offending implant, treatment was limited to specific features, such as strabismus surgery for esotropia and drainage for hematomas [10,12,13]. When esotropia remains despite implant removal, strabismus surgery should be considered as it resulted in improvement, although not resolution, of symptoms in all three cases [10,12,13].

Other Ocular Complications

Zygomatic fistulation can occur secondary to zygomatic implants as a late-onset complication occurring months after implantation [9,14,25]. Zygomatic implants can be a nidus for infection and inflammation, particularly when the implant penetrates through the zygomatic bone into subcutaneous tissue [6,9,31]. Implants should be placed with minimal penetration through the zygomatic bone to avoid complications, with greater penetration rectified by performing an implant apicoectomy [6,31]. Alternatively, improper irrigation during surgery can lead to retained contaminated debris from the oral mucosa, resulting in chronic infection [9]. Periorbital infection and fistulation can present with erythema, suppuration, and conjunctival chemosis. Treatment for fistulation includes antibiotics, implant apicoectomy, and fistulectomy reserved for refractory cases [9]. Resolution of fistulas occurred in all reported cases following treatment, although one case reported contralateral fistulation months later [9].

Periorbital hematomas are commonly seen after a zygomatic implant procedure. Although only four cases are reported in the literature, this is likely due to their expected occurrence and benign course. The one study that reported periorbital hematomas found a 40% (4/10) incidence rate [21]. All four cases occurred immediately or soon after surgery and resolved within one week. 

Temporary infraorbital paresthesias are a common complication of zygomatic implantation. The paresthesia is usually self-limiting lasting between one week and three months after surgery [24,25]; however, some cases may be permanent [20]. Davó et al. [24] found all 21 cases performed in one surgical center experienced some infraorbital paresthesia, while none did in other locations. They postulated that the use of wider mucoperiosteal flaps was used in this location to gain a better view of the maxilla and infraorbital region, which may have contributed to the high rate of infraorbital paresthesias. All patients in this study regained full sensation without paresthesias within three months after surgery. 

## Conclusions

Zygomatic dental implants are an effective method of securing dental prostheses for patients with severe maxillary atrophy; however, serious ocular complications can occur. The most common complication is infraorbital paresthesia, which often self resolves. Orbital penetration is the most severe complication and can result in permanent ocular range of motion restriction, esotropia, and diplopia. Other complications include hematomas, emphysema, infraorbital nerve damage, fistulation, and infection. Therefore, zygomatic implantation should only be undertaken by experienced surgeons trained in the procedure. Although multiple methods have been proposed to decrease the risk of ocular complications, including steel plate placement, extra sinus technique, zygomatic orbital floor measurements, and dynamic navigational systems, further research is necessary to test the efficacy of these methods in preventing orbital complications.
